# Examination of Surgeons

**Published:** 1898-05

**Authors:** 


### Examination of Surgeons.

Some excitement in medical circles has been occasioned by the failure of several well known medical gentlemen of this State to pass the test examination, which is a pre-requisite to entering the United States Volunteer Service as surgeon.

